# Immunotherapy of prostate cancer using novel synthetic DNA vaccines targeting multiple tumor antigens

**DOI:** 10.18632/genesandcancer.214

**Published:** 2021-03-22

**Authors:** Devivasha Bordoloi, Peng Xiao, Hyeree Choi, Michelle Ho, Alfredo Perales-Puchalt, Makan Khoshnejad, J. Joseph Kim, Laurent Humeau, Alagarsamy Srinivasan, David B. Weiner, Kar Muthumani

**Affiliations:** ^1^Vaccine & Immunotherapy Center, The Wistar Institute, Philadelphia, PA, USA; ^2^Inovio Pharmaceuticals, Plymouth Meeting, PA, USA; ^3^NanoBio Diagnostics, West Chester, PA, USA; ^4^GeneOne Life Science Inc., Seoul, Korea; ^*^authors contributed equally

**Keywords:** prostate cancer, synthetic DNA vaccines, immune responses

## Abstract

Prostate cancer is a prevalent cancer in men and consists of both indolent and aggressive phenotypes. While active surveillance is recommended for the former, current treatments for the latter include surgery, radiation, chemo and hormonal therapy. It has been observed that the recurrence in the treated patients is high and results in castration resistant prostate cancer for which treatment options are limited. This scenario has prompted us to consider immunotherapy with synthetic DNA vaccines, as this approach can generate antigen-specific tumor-killing immune cells. Given the multifocal and heterogeneous nature of prostate cancer, we hypothesized that synthetic DNA vaccines targeting different prostate specific antigens are likely to induce broader and improved immunity who are at high risk as well as advanced clinical stage of prostate cancer, compared to a single antigen approach. Utilizing a bioinformatics approach, synthetic enhanced DNA vaccine (SEV) constructs were generated against STEAP1, PAP, PARM1, PSCA, PCTA and PSP94. Synthetic enhanced vaccines for prostate cancer antigens were shown to elicit antigen-specific immune responses in mice and the anti-tumor activity was evident in a prostate tumor challenge mouse model. These studies support further evaluation of the DNA tools for immunotherapy of prostate cancer and perhaps other cancers.

## INTRODUCTION

Prostate cancer (Pca) represents the most prevalent cancer type among males across the globe [1, 2]. It is the second leading cause of mortality due to cancer among American men and accounts for an estimated 191,930 cases diagnosed in 2020 with 33,330 deaths [3]. There have been important advances in management of prostate cancer. Treatment modalities such as surgery, radiation, chemo and hormone therapies have improved outcomes in patients with early-stage PCa. However, recurrence and progression of disease has been reported in 30-40% of patients who undergo radical prostatectomy, as defined by increased prostate specific antigen (PSA) levels in sera. Therapy for advanced stages of this disease presents a major challenge [4]. This supports advancing novel therapeutic strategies for improving management of PCa. Several recent studies suggest that immunotherapy for the induction of immune responses in PCa is among a handful of highly promising therapeutic strategies to be further advanced [5-7]. The determination of suitable antigens with expression confined to relevant tumors and high potential for immunogenicity in human, still remains a challenge [8, 9]. 

The prostate cancer full length antigens investigated in detail have focused on prostate-specific membrane antigen (PSMA), PSA and prostatic acid phosphatase (PAP) expressed through viral vectors, DNA vaccines, and personalized peptide vaccines [10, 11]. Cell therapies such as CAR (chimeric antigen receptor) - T cells against PSMA was also studied [12]. Different checkpoint inhibitors, bispecific antibodies and oncolytic viruses have also been investigated as immunotherapeutics for PCa [12]. In addition, vaccination with messenger RNA (mRNA) encoding distinctive tumor antigens which activates CD4 and CD8 cells was also investigated in PCa. For instance, Curevac introduced RNActive, containing both free and protamine-bound mRNA directed to 4 different antigens such as PSA, PSMA, PSCA and STEAP1, which demonstrated an immunological response through activation of B and T cells for all those antigens [12]. 

**Figure 1:**
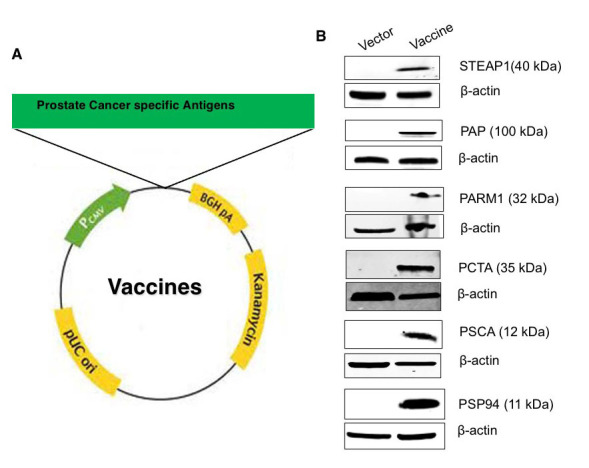
Design, generation, characterization, and expression analysis of Prostate Cancer Antigens (PCaA) SEV constructs. **(A)** Schematic representation of the PCaA-SEV construct generated. **(B)** Western blot analysis of SEV of STEAP-1, PAP, PARM1, PCTA, PSCA and PSP94. Human 293T cells were transfected with 2μg of each DNA vaccines or pMV101 and the cell lysates were collected after 48 h. 25μg of each lysate was then separated using polyacrylamide gel electrophoresis and subsequently transblotted; followed by the incubation with respective primary and then secondary antibodies. Cell lysates transfected with different PCaA- SEV revealed the correct molecular sized bands corresponding to the expression of respective proteins; however, no bands were present in pMV101 lanes. β-actin was used as loading control in all the cases.

Importantly, vast majority of the studies with these antigens have been carried out using single antigen. Considering the heterogeneous nature of prostate tumors, single antigen approach may not yield optimal immunity against cancer. Based on this information, we have hypothesized that inclusion of multiple tumor antigens as vaccine candidates would elicit an optimal response. In our view, targeting multiple antigens will confer better protection in comparison to single antigen alone. In the present study, we have utilized a Synthetic Enhanced DNA. Vaccine (SEV) platform to target multiple prostate cancer antigens. Both bioinformatic approaches and literature knowledge were utilized to select the SEV candidates. These include six‐transmembrane epithelial antigen of the prostate‐1 (STEAP1), prostatic acid phosphatase (PAP), prostate androgen regulated mucin-like protein 1 (PARM1), prostate carcinoma tumor antigen-1 (PCTA), prostate stem cell antigen (PSCA), and prostate secretory protein of 94 amino acids (PSP94). Our group has developed a synthetic consensus strategy where gene sequences from various species are compared to determine a consensus sequence exhibiting significant homology. Notably, this approach was found to break tolerance capacity, while retaining T cell killing against native MHC class I-presented sequences [13]. The 6 human genes; STAEP1, PAP, PARM1, PSCA, PCTA and PSP94 presented in this study share high homology with mouse, in fact 60-92% identity as revealed by Homologene, NCBI data base. We have evaluated immune responses as well as demonstrated the anti-tumor activity of these novel therapeutics in prostate specific tumor challenge model. The results show that targeting multiple prostate cancer antigens is an important strategy against PCa and SEV is an attractive immunotherapeutic approach against cancer owing to its safety, simplicity and stability [8, 14-17].

## RESULTS

### Generation and characterization of Prostate Cancer Antigens (PCaA-SEV) 

The selection of candidate tumor antigens was based on the information available from the published papers as well as our own analysis of the existing databases on PCa. The criteria we have used include overexpression of a gene in prostate tumors in comparison to normal prostate tissues or cells. In addition, overexpression of genes in relation to the early vs. advanced stage of disease was also taken into account. These efforts led us to select STEAP1, PAP, PARM1, PSCA, PCTA and PSP94 for our studies. Synthetic full-length gene sequences of STEAP1, PAP, PARM1, PSCA, PCTA and PSP94 were generated (Figure 1A) and cloned successfully using bioinformatics and synthetic DNA technologies as described earlier [8, 14, 18]. For validating the prostate-specific proteins as target antigens for vaccination, we initially checked for their expression by Western blot analysis using the lysates of cells transfected with PCaA-SEV. The results showed that lysates from HEK293T cells transfected with PCaA-SEV revealed the correct molecular sizes corresponding to the expression of each PCaA protein. Expression of β-actin served as endogenous control (Figure 1B). Together, successful generation of six different PCaA-SEV and protein expression in cells were confirmed to proceed with further studies. 

### Effect of PCaA-SEV immunization on antigen specific cellular immune responses 

**Figure 2:**
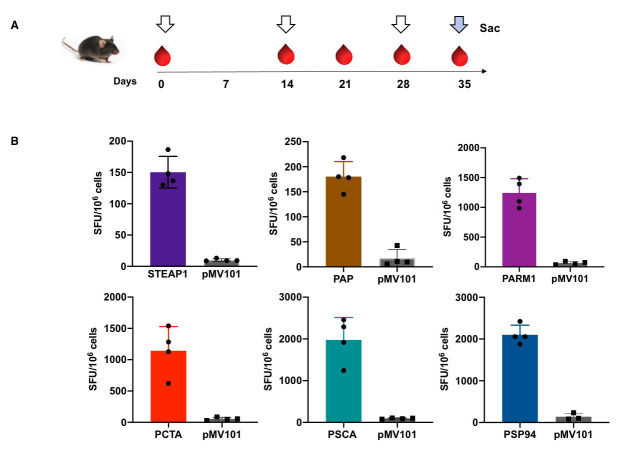
PCaA-SEV induces antigen specific cellular immune responses in mice. **(A)** Schematic representation of different time points of EP mediated immunization and immune analysis of this study. C57BL/6 mice were immunized with 50μg of PCaA-SEV or pMV101 using EP mediated enhanced delivery. A week after the third immunization (day 35), mice belonging to all the groups were euthanized and splenocytes were collected for ELISpot assay. **(B)** IFN-γ ELISpot assay, which was performed on splenocytes obtained from mice immunized with PCaA-SEV through EP after ex vivo stimulation with PCaA specific peptides. IFN-γ produced by the cells specific to these antigens are reported as spot forming units (SFUs) per million cells. In case of different PCaA vaccine groups, notably higher cellular immune responses were found to be generated compared to pMV101 group of mice. The graphs represent average IFN-γ SFUs generated per 10^6^ splenocytes +/- SEM for the target peptide. Group average spot forming units (SFU) per million cells are presented.

The immunization strategy adapted for vaccination dosage is presented in Figure 2. Mice (C57BL/6, male) were grouped and immunized with 50μg of PCaA-SEV or pMV101 vector control followed by electroporation (EP) to enhance DNA delivery. In order to assess vaccination induced interferon gamma (IFN-γ) -producing T cells, ELISpot assays were performed using the spleen cells isolated from mice immunized with PCaA-SEV or pMV101 empty vector after stimulating with specific peptides [17]. As shown in the schematic representation (Figure 2A), a week after the third immunization (day 35), bulk splenocytes from mice immunized with the PCaA-SEV were obtained for ELISpot assay. Briefly, splenocytes from mice were ex vivo stimulated with PCaA peptides. IFN-γ produced by the cells specific to the antigens are reported as spot forming units (SFUs) per million cells (Figure 2B). Notably, mice immunized with PSP94 DNA vaccine exhibited the most robust cellular responses. Similarly, PSCA, PCTA and PARM1 vaccine candidates also showed robust cellular responses to antigens. The splenocytes from mice immunized with STEAP1 and PAP-SEV registered low level of cellular immune responses compared to other vaccines candidates. Collectively, these data demonstrated that PCaA-SEV induced antigen specific cellular immune responses in mice. 

### PCaA-SEV generated polyfunctionality in both CD4^+^ and CD8^+^ T cells 

**Figure 3:**
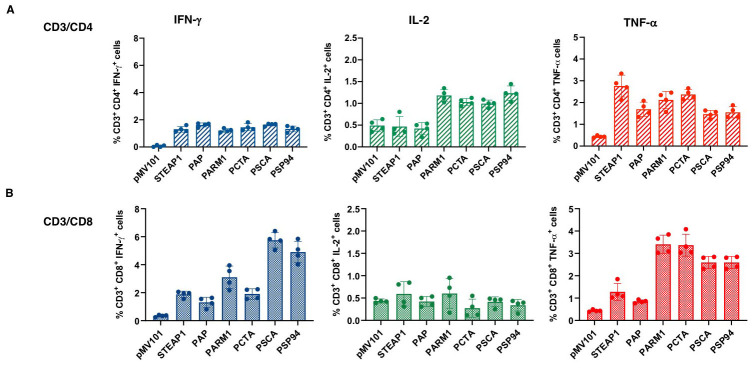
PCaA-SEV induces both CD4^+^ and CD8^+^ T cell responses in mice. Splenocytes from PCaA-SEV immunized mice as shown in the previous experiments were also evaluated by polychromatic flow cytometry to measure **(A)** CD4^+^ and **(B)** CD8^+^ T cells producing different cytokines. Splenocytes obtained from mice, after three immunizations of PCaA-SEV or pMV101, were stimulated with respective PCaA target peptides *ex vivo* and then stained with different fluorophore-tagged antibodies as shown, for determining the production of cytokines by both CD4^+^ and CD8^+^ T cells. Graphs indicate the total percentage of IFN-γ^+^, TNF-α^+^, and IL-2^+^ T cells (mean ± SEM). PCaA-SEV resulted in higher frequency of CD4^+^ as well as CD8^+^ cells secreting intracellular cytokines upon ex vivo stimulation with antigen specific peptides.

T cell polyfunctionality refers to the single-cell level co-expression of multiple functional molecules [19]. Upon understanding the potent PCaA-SEV induced immune responses through IFN-γ ELISpot assay, we further determined the overall immunomodulatory effects of PCaA-SEV through staining of intracellular cytokines to evaluate the character of distinct functional CD8^+^/CD4^+^ T cell populations. For this purpose, splenocytes from C57BL/6 mice receiving three immunizations of PCaA vaccines or pMV101 were evaluated with the help of polychromatic flow cytometry. Specifically, bulk splenocytes were stimulated with vaccine specific PCaA peptides *ex vivo*. After permeabilization and fixation, cells were stained intracellularly with different fluorophore-tagged antibodies against IFN-γ, tumor necrosis factor-α (TNF-α), and interleukin 2 (IL-2). Stained cells were acquired using a LSR-II flow cytometer and data were analyzed described in the Materials and Methods, for determining CD4^+^ (Figure 3A) as well as CD8^+^ T cells production (Figure 3B) of the activated-state cytokines including IFN-γ, TNF-α, and IL-2. It was observed that mice immunized with different PCaA-SEV exhibited higher frequency of CD4^+^ T cells secreting each intracellular cytokine upon stimulation with PCaA peptides. Similarly, CD8^+^ T cells isolated from the mice vaccinated with PCaA-SEV were also found to produce IFN-γ and TNF-α post PCaA peptides’ stimulation. Thus, PCaA-SEV were noted to induce both cellular immunity to PCaA as well as polyfunctionality of antigen-specific T cells. 

### PCaA-SEV Vaccine induced humoral immune responses 

**Figure 4:**
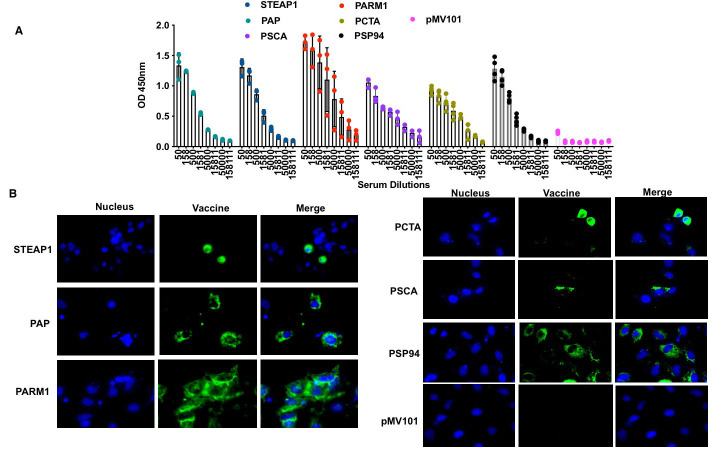
PCaA-SEV induces humoral immune response. **(A)** ELISA reactive antibodies following the third dose of immunization with PCaA-SEV (day 35). Sera were diluted as shown and vaccine-specific IgG reacting with each antigen was determined through ELISA. Mean optical density and SEM for each group/ dilution against each antigen is indicated. **(B)** Indirect immunofluorescence analysis of prostate antigen expression in HepG2 cells expressing different PCaA-SEV to confirm whether antibodies induced by the experimental prostate antigen could recognize vaccine-transfected cells. 48hrs post transfection of HepG2 cells, incubated with pooled day 35 sera (1:100) from mice immunized with different PCaA-SEV (50ug/immunization); Alexa Fluor 488-tagged anti-mouse IgG secondary antibody (green) and DAPI (blue) were used in this assay.

Next, we investigated the humoral immune responses of the PCaA-SEV. Firstly, we determined the antigen-specific antibody responses induced by each vaccine. Mice were immunized with the specific antigen and individual sera were collected for evaluating the reactivity of IgG antibodies in immune sera by ELISA (Figure 4A). PCaA-SEV immune sera showed reactivity to the target antigen. Further, sera collected at day 35 were also tested by an immunofluorescence assay (IFA) to determine whether immune sera could recognize the production of specific antibodies against the target antigen (Figure 4B). These data indicated that each of the individual components induced a humoral immune response, accompanied by strong binding. Importantly, these data also demonstrated that the synthetic DNA immunization along with electroporation induced a balanced antibody response similar to that induced by the cellular responses. 

### Intramuscular administration of the PCaA-SEV elicited antitumor immunity against prostate cancer 

**Figure 5:**
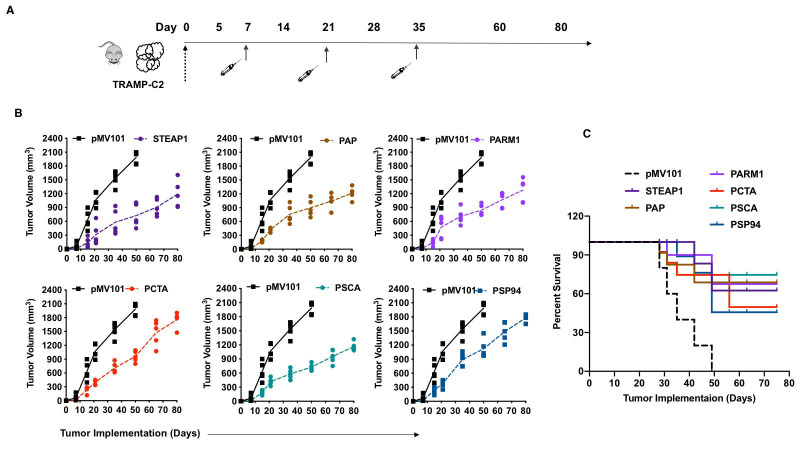
PCaA-SEV delays tumor progression and enhances survival of prostate cancer bearing mice. **(A)** Schema of TRAMP-C2 tumor cells administration and pMV101 or PCaA-SEV administration into C57BL/6 mice. Mice were administered subcutaneously 1.0 x 10^6^ TRAMP-C2 cells. After TRAMP-C2 tumor challenge in C57BL/6 mice on day 0, mice were immunized with PCaA-SEV (50μg/immunization) on day 7, 21 and 35 through optimized EP enhanced delivery. **(B)** Assessment of tumor development in control plasmid (pMV101) and PCaA-SEV+TRAMP-C2 cells injected mice. Tumor volumes (mm^3^) were measured weekly, by a digital caliper, for up to 80 days post tumor administration in mice. Mice inoculated with PCaA-SEV plasmid exhibited delayed tumor growth, as evinced by tumor volume. (C) Kaplan–Meier survival curves of TRAMP-C2 prostate tumor bearing mice immunized with PCaA-SEV or pMV101 vector. Mice immunized with PCaA-SEV were found to exhibit improved survival compared to the pMV101 vaccinated mice.

The assessment of the ability of vaccine-induced tumor-specific responses to provide protection against the disease in an animal challenge model is highly critical for establishing the therapeutic efficacy of any vaccine. We, therefore, evaluated the potential of PCaA-SEV by determining their ability to inhibit the growth of established prostate tumors. The schematic representation of the immunization as well as challenge strategies for the TRAMP-C2 mice model is shown (Figure 5A). C57Bl/6 mice were inoculated with TRAMP-C2 PCa cells as mentioned in the materials and methods section. Seven days post inoculation with the PCa cells, the tumors were noted to be palpable. Then, the different groups of mice were immunized with 50μg of PCaA-SEV or pMV101 vector, intramuscularly once weekly starting on day 7 for a total of three immunizations through EP-mediated delivery. Notably, vaccination with the different PCaA-SEV led to the delayed tumor progression in mice in comparison with the pMV101-vaccinated group (Figure 5B). Thus, PCaA-SEV vaccination through EP enhanced delivery exerted potent effect against prostate tumor in TRAMP-C2 mice model, which was well evinced from the long-term survival of the PCaA vaccinated mice compared to the pMV101 vaccinated ones. (Figure 5C). 

**Figure 6:**
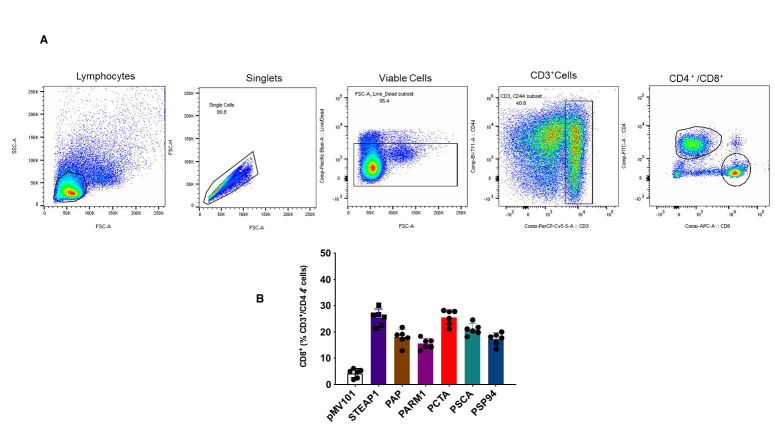
PCaA-SEV promotes T cell recruitment to the tumor microenvironment. Mice were immunized with PCaA-SEV, 3 times at 2-week intervals and challenged with TRAMP-C2. Three weeks after tumor inoculation, a paracentesis was performed for analysis of leukocyte subsets by flow cytometry. **(A)** Flow cytometric representation of CD8^+^ T cells from the total CD3^+^ and CD44^+^ cells. **(B)** Enhanced infiltration of anti-tumor CD8^+^ T cells in the tumor microenvironment of mice vaccinated with PCaA-SEV, after inoculation with TRAMP-C2 prostate tumors for 3 weeks.

Further, we determined the percentage of CD8^+^ T cells of the total CD3^+^ and CD45^+^ cells in the tumor microenvironment in the PCaA-SEV vaccinated group of mice, 3 weeks after tumor inoculation (Figure 6A). Upon analysis, an increase in T cell response against the prostate tumor was detected in the tumor microenvironment of vaccinated mice groups (Figure 6B). Thus, PCaA-SEV vaccination led to enhanced infiltration of anti-tumor CD8^+^ T cells in the tumor microenvironment. Taken together, our findings suggested that EP mediated enhanced delivery of these DNA vaccines were able to generate PCaA specific CD8^+^T cells and elevate their levels in the tumor microenvironment leading to improved survival of the mice bearing prostate tumor. 

## DISCUSSION

Cancer immunotherapy has emerged as a breakthrough treatment modality for diverse malignancies, through the use of cancer vaccines, immune checkpoint inhibitors, adoptive cell therapy. Presently, different cancer vaccine platforms such as peptide and recombinant virus vector-based vaccines, dendritic cell vaccines, engineered cellular vaccines, and idiotype vaccines have been established [20]. In addition, recently emerged DNA vaccines represent another platform for treating different pathogens and evasive diseases including cancer [15, 21, 22]. DNA vaccines are highly flexible and versatile as they offer easy manipulation of vaccine targets through alteration of gene sequences of the delivered plasmid DNA [23]. Furthermore, these vaccines have the ability to induce potent antitumor cell-mediated immune responses against a diverse range of tumor antigens [24]. Although, there exist different tumor-specific antigens with unique expression on a lineage of distinct tumor cells, identifying the suitable tumor-specific antigens to develop targeted therapy, causing minimal impairment to the normal cells, is still a challenge [24, 25]. STEAP1, PAP, PARM1, PCTA, PSCA, and PSP94 are different prostate specific proteins which are found to be expressed in normal as well as malignant prostatic cancer tissues. STEAP1 is a cell surface protein, primarily located at cell-cell junctions, which is found to have limited expression in normal tissues, whereas high expression in primary PCa tissues [26]. Increasing lines of evidence suggest STEAP1 as an effective biomarker and a potent target antigen for immunotherapy against prostatic malignancy [27]. Cytotoxic T lymphocytes (CTLs) specific to STEAP1 led to the inhibition of transplantable prostate tumor cells’ growth *in vivo* [27-29]. Another prostate tumor antigen, PAP is the target of Sipuleucel-T, the FDA-approved anti-tumor vaccine [30]. It is a secretory prostate-specific protein consisting of 354 amino acids. Over 95% of PCa tissues exerted elevated expression of PAP [31]. PARM1 codes for a 298-amino acid protein. Although low-level of PARM1 expression is detected in other organs besides prostate, its regulation by androgens seems to be limited to this gland [32]. It was initially known as a highly induced gene in the prostate, post castration in rats. Elevated rat PARM1 expression was reported to cause enhanced telomerase function and the immortalization of prostate cancer cell lines, implying its role in the regulation of prostate cells’ survival [33]. Further, PCTA is another surface marker, found to be strongly linked with PCa [34]. It encodes a 35kDa secreted protein having around 40% sequence homology with the N-amino terminal region of the S-type galactose-binding lectin (galectin) gene family members which are known to play role in tumorigenesis and metastasis [35]. PSCA, a glycosylphosphatidylinositol (GPI)-anchored cell surface protein is a newly identified tumor associated antigen which functions as an important marker for PCa [36]. This protein is reported to exhibit elevated expression level in above 80% local PCa cases and in all bone metastatic lesions [37]. Notably, PSCA is considered as an effective marker for late stage PCa as its overexpression possess strong correlation with advancing tumor grade, stage, and progression to androgen independence [37]. In addition, it anchors to cancer cell surface without exocytosis and therefore it is considered as a highly suitable target antigen for PCa immunotherapy [38]. PSP94, also known as prostatic inhibin or β-micro semino protein is one of the most abundant proteins in semen along with PSA and PAP. As with other prostate-secreted proteins, PSP94 can leak into the blood upon benign or malignant prostate epithelial disruption and can be measured within serum. PSP94 was previously studied as a PCa blood biomarker [39, 40]. Consequently, targeting these proteins can provide a new avenue for developing anti-tumor vaccines against PCa. 

Notably, a number of pre-clinical and clinical studies have evaluated the role of these prostate specific antigens. For instance, Moreaux and group reported that immunization with STEAP1 antigen through modified vaccinia virus Ankara vector in a murine subcutaneous tumor model led to the marked inhibition of PCa progression [41]. Further, a renewed interest was generated in PAP due to its ability to predict intermediate to high-risk PCa cases and its success in PCa immunotherapy [42]. PAP-specific T-cell responses were reported to elicit and augment in human as well as animal models after antigen-specific immunization [43, 44]. Yang and colleagues showed a PAP encoded DNA vaccine to result in PAP-specific CD8^+^ T cell immune responses. Additionally, a phase I/II trial was conducted using a DNA vaccine encoding human PAP to treat 22 stage D0 PCa patients. The findings revealed PSA values not to be dropped by more than 50% in the patients following treatment, however some patients were found to exhibit reduction in serum PSA rise rate [45]. Furthermore, PARM1 enabled the prostate cells to resist apoptosis via increased telomerase function [32, 46]. 

In addition, anti-PSCA CAR-T cells have been considered to have potential to treat metastatic PCa [47]. Besides, PCTA is speculated to contribute as a low risk factor to the susceptibility of PCa in sporadic disease [48]. PSP94 was reported to play role in growth regulation and apoptosis induction in PCa cells. They are known to regulate the levels of calcium during the hypercalcemic condition of malignancy [39]. Further, its expression in radical prostatectomy tumor specimens was seemingly found to be linked with poor survival and thus signifies its potent prognostic importance [49]. In this study, we evaluated the efficacy of these PCaA-SEV in the pre-clinical setting. We firstly synthetically designed full-length gene sequences of different prostate specific antigens namely STEAP1, PAP, PARM1, PSCA, PCTA and PSP94. They were then successfully transfected, and their expressions were confirmed in HEK293T cells through Western blot analysis. The assessment of the PCaA vaccines demonstrated induction of cellular immunity as well as polyfunctionality of antigen-specific T cells. The highest cellular responses were observed with PSP94 DNA vaccine. In addition, PCTA and PSCA vaccine groups also exhibited markedly robust cellular immune responses. PCaA-SEV resulted in higher frequency of CD4^+^ and CD8^+^ T cells secreting intracellular cytokines. Further, the consensus sequences generated by these individual prostate antigens were capable of generating potent humoral immune responses to each antigen. Additionally, PCaA-SEV through EP mediated delivery was found to delay tumor progression and cause enhanced infiltration of anti-tumor CD8^+^ T cells in the tumor microenvironment, resulting in the long-term survival of the TRAMP-C2 mice and thus provided protection from prostate tumor. 

The results presented here suggest that the selection of genes, based on multiple criteria including expression pattern, as candidate vaccines combined with the advances in DNA delivery technologies provide a valuable immunotherapeutic approach to treat prostate cancer. Specifically, there is a need for novel treatment options for individuals who show recurrence of PCa after undergoing surgery and radiation treatments for early-stage cancer and also individuals with advanced stage of cancer. These groups would greatly benefit from immunotherapeutic approaches [10, 50]. Another advantage with this strategy is the ability to combine vaccine candidates for simultaneous attack on multiple targets to suppress the tumor growth. Further, the immunotherapeutic approach can also be combined with other treatment modalities such as chemo and radiation therapies for the effective management of PCa. Therefore, additional studies are highly warranted to fully establish the clinical significance of these synthetic DNA vaccines targeting PCaA, which in turn could result in the better clinical management of this neoplasm. 

## MATERIALS AND METHODS

### Cell lines and reagents 

HEK293T, HepG2 and TRAMP-C2 cells were procured from ATCC. These three cell types were maintained in D10 media: Dulbecco’s Modified Eagle Medium (Invitrogen Life Science Technologies, San Diego, CA, USA) supplemented with 10% heat-inactivated fetal calf serum (FCS), 3 mM glutamine, 100 U/ml penicillin, and 100 U/ml streptomycin. For mouse splenocyte cells, R10 media: (RPMI1640, Invitrogen Life Science Technologies, San Diego, CA, USA) supplemented with 10% heat- inactivated FCS, 3 mM glutamine,100 U/ml penicillin, and 100 U/ml streptomycin was used. All the cells were maintained and grown in a 5% CO_2_ regulated incubator set at 37 °C [18]. 

### Construction of prostate cancer antigens- synthetic enhanced DNA vaccine (PCaA-SEV) 

Sequences of human prostate cancer antigens (PCaA) such as STEAP1, PAP, PARM1, PCTA, PSCA, and PSP94 were retrieved from NCBI database and immunogens were designed using codon- and RNA-optimized method as described before [8, 17, 19, 51]. Further, they were cloned individually into a pMV101 vector (GenScript, Piscataway, NJ, USA) under the control of the cytomegalovirus immediate-early promoter [8, 14, 18]. The SEV expressing STEAP1, PAP, PARM1, PCTA, PSCA and PSP94 are designated as STEAP1 vaccine, PAP vaccine, PARM1 vaccine, PCTA vaccine, PSCA vaccine, and PSP94 vaccine respectively and together referred as PCaA-SEV henceforth. 

### Transfection and expression of PCaA-SEV 

HEK293T cells were seeded at a density of 6x10^5^ cells/well in six-well plates. After 24 hours, the cells were transfected with the above mentioned PCaA plasmids as well as pMV101 control plasmids using GeneJammer transfection reagent (Agilent Technologies, Santa Clara, CA, USA) as per the manufacturer’s protocol. After 48 hours, the lysates of the transfected cells were collected, and Western blot analysis was performed for validating the antigen expression. Cell lysis was carried out using lysis buffer (50 mM HCl, 150 mM NaCl, 1% Nonidet P-40, 1% Triton X-100, 0.1% sodium dodecyl sulfate), and a cocktail of protease inhibitors (Roche, Basel, Switzerland). The supernatants obtained after cell lysis were then analyzed using sodium dodecyl sulfate-12% polyacrylamide gel electrophoresis. Subsequently, they were transferred to a polyvinylidene difluoride (PVDF) membrane and then incubated with primary antibodies against PCaA i.e. anti-STEAP, anti-PCTA, anti-PSP94 (R&D Systems, Minneapolis, MN, USA); anti-PAP (Cell signaling Technology, Danvers, MA, USA); anti-PARM1, and anti-PSCA (ThermoFisher, Waltham, MA, USA). Following this, the membrane was incubated with appropriate horseradish peroxidase (HRP) conjugated secondary antibodies (Li-Cor, Nebraska, USA). Then, the stripping of the membrane was done with the help of NewBlot Nitrocellulose 5x stripping buffer (Li-Cor, Nebraska, USA) followed by probing with β-actin (Li-Cor, Nebraska, USA). β-actin served as the loading control. 

### Enzyme Linked Immunosorbent Assay (ELISA) 

For binding ELISA, firstly MaxiSorp high-binding 96-well ELISA plates (ThermoFisher, USA) were coated with different recombinant antigens at a concentration of 1μg/mL in PBS at 4°C overnight. The plates were then washed 4 times with PBS containing 0.01% Tween-20 (PBST). Subsequently, blocking was done with 10% FBS in PBS for 1 hour at 37°C. Serum samples (collected from mice immunized with 50µg of PCaA-SEV at day 35) were serially diluted (starting at 1:50, dilution factor 3.16) in PBS with 1% FBS. Then 100μl of the diluted serum samples were added to the wells and incubated for 2 hours at 37°C. Following the incubation, the plates were washed 4 times with PBST and incubated with HRP-conjugated goat anti-mouse IgG (Sigma-Aldrich, St. Louis, MO, USA) for 1 hour at 37°C. Then 100μl of 3,3’5,5’-Tetramethylbenzidine (TMB) Substrate (Sigma-Aldrich, St. Louis, MO, USA) was added to each well after the final wash and incubated for 10 minutes. The reaction was stopped by addition of 100μl of 2N H_2_SO_4_ per well. Finally, the optical density of the plate was measured at 450 nm using an ELISA plate reader (Biotek, Winooski, VT, USA). 

### Immunofluorescence Analysis 

In case of IFA, HepG2 liver cancer cells were seeded in 6-well cell culture plates on coverslips followed by transfection with PCaA-SEV as well as pMV101 empty vector as discussed [19]. The cells were then incubated with sera collected from mice immunized with 50µg of PCaA-SEV at day 35. Nuclear staining was done with 4′, 6-diamidino-2-phenylindole (DAPI) by incubating for 20 minutes at room temperature. Further, PCaA proteins were stained with the immunized sera (1:100) and then incubated with Alexa Fluor 488 dye. After each incubation step, washing with Phosphate-buffered saline (PBS) was carried out. Finally, the samples were mounted onto glass slides with the help of Fluoroshield mounting medium (Abcam, Cambridge, MA, USA) and then observed under a microscope (Eclipse 80i, Nikon). 

### Animals, Study approval, Plasmid administration and EP delivery 

Male C57BL/6 mice (five- to eight-week-old) were procured from the Jackson Laboratory, ME, USA and vaccinated in a light-cycled, temperature- and humidity-controlled animal facility. All aspects of the experimental design and procedure were reviewed and approved by the institutional ethics and animal welfare committees of the Wistar Institute (Protocol #112767). The mice were separated into different groups and immunized with 30μl of 50μg pMV101 and 50μg of different PCaA-SEV, intramuscularly, thrice at the intervals of 2-week followed by EP (CELLECTRA; Inovio Pharmaceuticals, Plymouth Meeting, PA, USA) [19]. Specific pulsing parameters used for delivery were 2 pulses of 0.1 Amp constant current, 4s apart and 52ms in length [52]. The mice were housed in a barrier animal facility at the Wistar Institute. 

### Isolation of splenocyte and Interferon‐gamma (IFN-γ) ELISpot assay 

The spleens of the mice were dissected and crushed using a Stomacher device (Seward, UK) and the splenocytes were filtered through a 40μm cell strainer (ThermoFisher, Waltham, MA, USA). For the lysis of red blood cells, the splenocytes were treated with Ammonium-Chloride-Potassium (ACK) lysis buffer (Quality Biologicals, MD, USA) for 5 minutes. Subsequently, Mouse IFN-γ ELISpot PLUS assay (Mabtech, Cincinnati, OH, USA) was carried out using the splenocytes resuspended in R10 as per the manufacturer’s protocol. Precisely, splenocytes from PCaA-SEV or pMV101 immunized mice were added at a density of 2x10^5^/well in plates and then incubated separately in the presence of only media (negative control), media along with cell activation cocktail (BioLegend, San Diego, CA, USA), pre-mixed phorbol 12-myristate-13-acetate (PMA) and ionomycin (positive control), and media with peptides with a final concentration of 1μg/ml, for 18 hours at 37°C in a 5% CO_2_ regulated incubator. PCaA-SEV derived synthetic peptides were synthetized by Genscript, USA. The peptides were dissolved in DMSO and stored at -80°C. Bioinformatics approach using the SYFPEITHI website (www.syfpeithi.com) was utilized to define the dominant epitopes. Subsequently, upon addition of 5-bromo-4-chloro-3-indolyl-phosphate/nitro blue tetrazolium (BCIP/NBT) color development substrate (R&D Systems, Minneapolis, MN, USA), formation of spots were observed and the spot forming units (SFU) were then quantified with the help of automated ELISpot reader (CTL Limited, Ohio, USA). 

### Flow cytometry and intracellular cytokine staining assay 

Mouse splenocyte cells were seeded at a density of 2x10^6^ cells/well in a U-bottom 96-well plate (ThermoFisher, Waltham, MA, USA). The cells were then stimulated in the presence of media alone (negative control), or media with Cell Activation Cocktail (BioLegend, San Diego, CA, USA) containing pre-mixed PMA and ionomycin (positive control), or with media containing different PCaA peptides (1μg/ml), where all the samples contained a protein transport inhibitor cocktail (eBioscience, San Diego, CA, USA) at 37°C for 5 hours in a CO_2_ regulated incubator. Following stimulation, the cells were washed with FACS buffer (PBS containing 0.1% sodium azide and 1% FBS) and then stained for the surface proteins using fluorochrome-conjugated antibodies. The cells were again washed with FACS buffer. Before staining with intracellular cytokines using fluorchrome-conjugated antibodies, cells were fixed and permeabilized with the help of BD Cytofix/Cytoperm (BD Biosciences, San Diego, CA, USA). Mouse antibodies used for staining in this assay were CD19 (V450; clone 1D3; BD Biosciences), CD3 (145-2C11; Biolegend), CD4 (RM4-5; eBioscience), CD8 (53–6.7; BD Biosciences), CD44 (IM7; BioLegend) interferon-γ (XMG1.2; Biolegend), TNF-α (MP6-XT22; eBioscience), and interleukin-2 (JES6-SH4; eBioscience). Live/dead exclusion was done with the Violet viability kit (Invitrogen Life Science Technologies, San Diego, CA, USA). All the data were acquired from an LSRII flow cytometer (BD Biosciences) and FlowJo software (Tree Star, Ashland, OR, USA) was used for analysis. 

### Tumor challenge: tumor inoculation and monitoring 

For the tumor challenge study, C57BL/6 male mice were inoculated with 1.0×10^6^ TRAMP-C2 cells (in 200μl PBS) subcutaneously in the right flank at day 0 followed by three vaccinations with PCaA-SEV or pMV101 at days 7, 21 and 35. Tumor masses were measured with a digital caliper, and tumor volumes were calculated approximating the tumor mass to a sphere, according to the following equation: {tumor volume = ½(length x width^2^)}. Moreover, the tumor-bearing mice were monitored daily for their survival. When the tumors obtained a size of 2 cm in diameter, they were humanely euthanized. 

### Statistics

All the statistical analyses were carried out with the help of GraphPad Prism software. Data are represented as the mean ± Standard Error of the Mean (SEM). A two-tailed t-test for studies with only 2 experimental groups and one-way ANOVA to test for experiments with more than 2 experimental groups were performed for determining the statistical significance.

## Abbreviations

SEV: Synthetic enhanced DNA vaccine
PSMA: Prostate-specific membrane antigen
PAP: prostatic acid phosphatase
PARM1: Prostate androgen regulated mucin-like protein 1
PCTA: Prostate carcinoma tumor antigen-1
PSCA: prostate stem cell antigen
PSP94: prostate secretory protein of 94 amino acids (PSP94)
PSA: Prostate specific antigen
CAR: Chimeric antigen receptor
MHC: Major histocompatibility complex
NCBI: National Center for Biotechnology Information
HEK293T: Human embryonic kidney 293T cells
IFN-γ: Interferon gamma
IFA: Immunofluorescence assay
IgG: Immunoglobulin G
TRAMP: transgenic adenocarcinoma of the mouse prostate
HepG2: hepatocellular carcinoma cell line
GPI: glycosylphosphatidylinositol
SEM: Standard error of the mean
